# Developmentally Restricted Genetic Determinants of Human Arsenic Metabolism: Association between Urinary Methylated Arsenic and *CYT19* Polymorphisms in Children

**DOI:** 10.1289/ehp.7780

**Published:** 2005-03-22

**Authors:** Maria Mercedes Meza, Lizhi Yu, Yelitza Y. Rodriguez, Mischa Guild, David Thompson, A. Jay Gandolfi, Walter T. Klimecki

**Affiliations:** ^1^Department of Natural Resources, Sonora Institute of Technology (ITSON), Ciudad Obregon, Sonora, Mexico;; ^2^Arizona Respiratory Center, and; ^3^Department of Pharmacology and Toxicology, University of Arizona, Tucson, Arizona, USA

**Keywords:** arsenic metabolism, *CYT19*, genetic association, *GSTO*, pharmacogenetics, *PNP*, polymorphism, SNP

## Abstract

We report the results of a screen for genetic association with urinary arsenic metabolite levels in three arsenic metabolism candidate genes, *PNP*, *GSTO*, and *CYT19*, in 135 arsenic-exposed subjects from the Yaqui Valley in Sonora, Mexico, who were exposed to drinking water concentrations ranging from 5.5 to 43.3 ppb. We chose 23 polymorphic sites to test in the arsenic-exposed population. Initial phenotypes evaluated included the ratio of urinary inorganic arsenic(III) to inorganic arsenic(V) and the ratio of urinary dimethylarsenic(V) to monomethylarsenic(V) (D:M). In the initial association screening, three polymorphic sites in the *CYT19* gene were significantly associated with D:M ratios in the total population. Subsequent analysis of this association revealed that the association signal for the entire population was actually caused by an extremely strong association in only the children (7–11 years of age) between *CYT19* genotype and D:M levels. With children removed from the analysis, no significant genetic association was observed in adults (18–79 years). The existence of a strong, developmentally regulated genetic association between *CYT19* and arsenic metabolism carries import for both arsenic pharmacogenetics and arsenic toxicology, as well as for public health and governmental regulatory officials.

Scientific effort focused on the characterization of the mechanisms of arsenic-induced human toxicity is currently proceeding along several lines of research, including characterization of the spectrum of disease manifestations in human arsenicism, discovery of the proximal biochemical targets of arsenic toxicity, and the elucidation of the gene products involved in the complex biotransformation of arsenic (Karagas et al. 2004; Thomas et al. 2004). Understanding the biotransformation of arsenic is essential to a complete understanding of human arsenicism—a point that is underscored by the wealth of arsenic toxicology literature suggesting that the various chemical forms of arsenic in the human biotransformation scheme can have markedly different toxic potencies and spectra of biologic targets (Mass et al. 2001; Styblo et al. 2000, 2002). This relationship between the production and clearance of various chemical forms of arsenic and the particular manifestation of arsenic toxicity in humans could explain both the variability in measured phenotypes relating to arsenic metabolism in exposed human populations and the long-standing observation of individual variability in arsenic toxicity among relatively homogeneously exposed human populations (Rahman et al. 2003; Watanabe et al. 2001). If the metabolism of arsenic follows similar parameters as the metabolism of a number of xenobiotics, genetic variation in metabolic pathway members could be a determinant of individual variation in metabolism and concomitant toxicity (Hein et al. 1992; May 1994). For some time now hypotheses have been advanced that invoke genetic determinants of individual variability in arsenic metabolism, based on the observed variation in urinary arsenic metabolic profiles in humans, and on interspecies and intraspecies strain differences in animal models (Styblo et al. 1999; Thomas et al. 2001; Vahter 2000). More recently, the case for genetic determinants of variability in human arsenic metabolism was strengthened by Chung et al. (2002), who found a stronger correlation in arsenic methylation-related phenotypes among siblings than among genetically unrelated individuals.

To date, the arsenic literature specifically supports three genes as being involved in arsenic biotransformation: purine nucleoside phosphorylase (*PNP*), glutathione-*S*-transferase omega (*GSTO*), and arsenic(III) methyltransferase (*CYT19*) (Lin et al. 2002; Radabaugh et al. 2002; Zakharyan et al. 2001). (Recently the HUGO-recognized name for *CYT19* has been designated *AS3MT*.) Some but not all reports show that *PNP* is capable of functioning in the reduction of arsenate to arsenite (Nemeti et al. 2003; Radabaugh et al. 2002). *GSTO* is capable of the reduction of monomethylarsenic(V) [MMA(V)] to monomethyl-arsenic(III) (Nemeti and Gregus 2004). *CYT19* was initially characterized as an arsenic methyltransferase in rodents that is capable of the methylation of inorganic arsenic to its monomethyl form, and of monomethylarsenic to its dimethylarsenic form (Lin et al. 2002). In rodents, *CYT19*, in the presence of the proper system of reducing equivalents, has been proposed to be capable of the entire gamut of arsenic biotransformations that begin with arsenite and end with dimethylarsenic(V) [DMA(V)] (Thomas et al. 2004). These studies have provided the identity of candidate genes and the basis for beginning the genetic association studies necessary to test the hypotheses of the existence of genetic determinants of interindividual variability of arsenic metabolism. A second prerequisite for genetic association studies is a catalog of the variable positions within the candidate genes, preferably ascertained in an ethnically relevant population. Collectively, several studies have produced these catalogs in various ethnic populations for *GSTO* (Marnell et al. 2003; Whitbread et al. 2003; Yu et al. 2003). In addition, catalogs of polymorphic sites in *PNP* have been published for European and indigenous Americans (Yu et al. 2003). Despite the availability of these catalogs, only one genetic association study has been reported (Marnell et al. 2003).

To address the need for genetic association studies aimed at testing the hypothesis of the existence of genetic determinants of interindividual variability in human arsenic metabolism, we used existing polymorphism catalogs for *GSTO* and *PNP*, produced a resequencing-derived catalog of polymorphisms in *CYT19* (no such resequencing-based catalog was publicly available), and tested 23 polymorphic sites within these three genes in a population of arsenic-exposed subjects from the Yaqui Valley area of Sonora, Mexico, who had been phenotyped for the levels of urinary metabolites of arsenic. We performed subsequent genetic association analysis to screen for the presence of statistically significant effects of genotypes on arsenic metabolism, as indirectly reflected by urinary arsenic metabolite levels.

## Materials and Methods

### Description of study subjects.

A total of 144 subjects were included in the study, ranging in age from 7 to 79 years, from several towns in the Yaqui Valley of Sonora, Mexico. Subjects were recruited in 2004 by contact through local health care officials, after attending an informational meeting in their hometowns. All subjects were in good health (self-reported and by physical examination) and free from any skin lesions suggestive of arsenic toxicity. Although we did not track the characteristics of individuals who declined to be in the study, participation rate was estimated to be > 90% of the individuals who attended the informational meetings. Before field collection, we decided to collect two age groups, an adult group ≥ 18 years of age and a child group between 7 and 11 years of age, with the lower age limit based on the logistics of sample collection and the upper age limit chosen to avoid confounding effects of puberty in the child group. The towns (and arsenic content of their drinking water) were Esperanza (each of two wells measured multiple times; combined mean ± SD, 43.3 ± 8.4 μg As/L), Cocorit (one well, 19.33 ± 0.8 μg As/L), Pueblo Yaqui (two wells, combined mean, 9.65 ± 0.23 μg As/L), Colonia Allende (one well, 5.5 ± 0.20 μg As/L), and Campo 47 (one well, 5.5 ± 0.07 μg As/L). Subjects reported that their sole source of drinking water was well water, although we did not independently validate this. Of the study population, 86 individuals were unrelated to any other study participants, 36 subjects consisted of duos of one parent and one child of that parent, 9 subjects consisted of trios of one parent and two children, 9 subjects consisted of trios of two parents and one child, and 4 subjects consisted of pairs of siblings. All subjects gave their informed consent, as approved by the Human Subjects Committee of the University of Arizona and the Ministry of Public Health of Sonora State. Participants were asked to exclude seafood from their diet for 3 days before sample collection. Physical data and data on health status, cigarette smoking, dietary habits, and other variables were obtained by questionnaire and physical examination. Subjects agreed to donate peripheral blood or buccal cells and urine samples for DNA isolation and arsenic determination, respectively. We failed to collect urine or tissue from five subjects, resulting in a total usable study population of 139 individuals.

### Tissue collection: blood samples.

Blood was collected by venous puncture. Personnel from the Ministry of Public Health withdrew 5 mL of blood from each subject into Vacutainers containing anticoagulant. The samples were transported on ice to the Sonora Institute of Technology, where they were kept at 4°C for up to 3 days before the DNA extraction was performed.

### Tissue collection: buccal samples.

Subjects vigorously rinsed their mouth twice, each time with 25 mL commercial mouthwash, and then discharged the rinse into a 50 mL conical tube. The tubes were stored at 4°C until the DNA extraction was performed.

### DNA isolation: blood samples.

Genomic DNA was extracted from whole blood samples using the Blood and Cell Culture Midi Kit (QIAGEN, Valencia, CA, USA).

### DNA isolation: buccal samples.

Tubes containing buccal cells were centrifuged at 4,200 rpm for 10 min, washed with sodium chloride solution, and centrifuged again at 4,200 rpm for 10 min. The cell pellet was then processed for DNA isolation using the QIAamp Mini kit (QIAGEN).

### Urine collection.

First morning void urine samples were obtained in 100-mL polypro-pylene bottles and kept on ice. Within 6 hr, cooled samples were taken to the Sonora Institute of Technology and kept frozen at −40°C. The accumulated samples were then shipped on dry ice to the University of Arizona, where the samples were stored at −80°C until the analysis was performed.

### Total arsenic analysis.

Urine samples were digested with nitric acid using a microwave oven, following published protocols (Francesconi et al. 2002). Freeze-dried urine reference material for trace elements (Clinchek-control; RECIPE, Munich, Germany), containing arsenic at a level of 68 μg As/L, was used for quality control and to validate the assay. Analysis of this standard by inductively coupled plasma/mass spectrometry (ICP/MS) yielded a range of 64.6–68.9 μg As/L with a range of recoveries of 94.9–101.3%.

### Arsenic speciation analysis.

Frozen samples were thawed at room temperature and diluted 2-fold using Milli-Q water and filtered with a 0.45 μm filter before injection (Mandal et al. 2001). The HPLC system consisted of an Agilent 1100 HPLC with a reverse-phase C18 column (Prodigy 3 μm ODS, 150 × 4.60 mm^2^; Phenomenex, Torrance, CA, USA), with an Agilent 7500 ICP/MS used as a detector for the analysis of arsenic species {(arsenic(III) [As(III)], arsenic(V) [As(V)], MMA(V), and DMA(V)}. The detection limits, quality control, precision, and accuracy of this analytical method were assessed. The detection limits were 0.42–1.08 μg/L for arsenic compounds. The precision was estimated 10 times with a solution containing approximately 10 times the detection limit concentrations, yielding percentages of relative standard deviation of 1.14–4.00%. Accuracy values were calculated by spiking standard compounds of all the species that were studied [10 μg/L As(III), As(V), and MMA(V); 20 μg/L DMA(V)] in urine samples. The recovery of the added compounds was 96–108%. Similar recovery experiments used reference urine samples (Institut National de Santéé Publique Québec, Québec, Canada), containing As(III), MMA(V), and DMA(V). Analyses of these standards yielded recoveries between 80 and 109%.

### Resequencing: resequencing subjects.

Anonymous DNA samples from healthy individuals of self-reported ancestry were obtained from the Coriell Institute (Camden, NJ, USA). We studied 22 samples from individuals of European ancestry (EA), and 24 samples from individuals of indigenous American ancestry (IA). EA individuals were selected from unrelated Centre d’Etudes du Polymorphisme Humain (CEPH; Paris, France) samples. The geographic origin of the IA samples consisted of five samples from Peru, nine samples from Mexico, one sample from Ecuador, and nine samples from Brazil. One DNA sample isolated from a chimpanzee was also included in the study. These samples are commercially available; sample identification and ordering information are available from the authors.

### Resequencing: PCR amplification.

Genomic sequence for *CYT19* was accessed from the Human Genome Browser (University of California, Santa Cruz 2004). Because of the large genomic size of human *CYT19*, we used a sampling strategy in determining the subset of the 32.4-kilobase (kb) genomic region to resequence. Included in the resequencing were approximately 2 kb 5′’ to the first exon, 1 kb 3′’ to the last exon, all exons, including at least 100 bases of the exon intron junction, and roughly evenly spaced regions of intron sequence. Within this strategy a total of 12.2 kb of the genomic region were resequenced. Polymerase chain reaction (PCR) amplicons were designed such that each amplicon was approximately 900 base pairs (bp) in length, and consecutive amplicons overlapped each other by approximately 200 bp. PCR reactions contained 20 ng genomic DNA, 1 pmol of each primer, 0.2 U Taq polymerase (platinum Taq; Invitrogen, Carlsbad, CA, USA), and 0.1 μM dNTPs in a total volume of 10 μL. Specific reaction conditions, including primer sequences, are available from the authors.

### Resequencing: direct PCR sequencing.

PCR amplicons were prepared for cycle sequencing by diluting them with water using a dilution range of 1:3 to 1:6, depending on the reaction yield, as determined by agarose gel electrophoresis. Cycle sequencing reactions were assembled using 0.4 μL of cycle sequencing premix (BigDye V3.0; Applied Biosystems, Foster City, CA, USA), 1 pmol of sequencing primer, 1.8 μL 5× sequencing dilution buffer and 5 μL of PCR product in a final volume of 10 μL. Cycle sequencing reactions were purified using DNA-affinity magnetic beads (Agencourt Biosciences, Beverly, MA, USA). Purified sequencing reactions were electrophoretically analyzed using a DNA Analyzer 3730xl (Applied Biosystems).

### Resequencing: polymorphism identification and analysis.

Sequence chromatograms were processed for base calling and assembly using the Phred, Phrap and Consed suite of software programs (Ewing et al. 1998; Gordon et al. 1998). Initial polymorphism tagging was performed using Polyphred, with a minimum sequence quality of phred 25 (Nickerson et al. 1997). Potential polymorphic sites that were initially identified by Polyphred were individually confirmed by visual inspection of sequence traces. A criterion of this visual inspection–confirmation was that the polymorphism must have been observed in multiple chromatograms from singleton polymorphisms (polymorphisms occurring in only one subject) or in multiple subjects. For these confirmed polymorphic sites, each genotype for each subject was also confirmed by visual inspection of chromatograms. Polymorphic sites and associated subject-identified genotypes were automatically output to a relational database for further analysis, which included the automated generation of ethnicity-specific genotype frequencies, allele frequencies, and goodness-of-fit tests for Hardy-Weinberg equilibrium. Haplotypes were inferred using a Gibbs-sampling algorithm as implemented in the PHASE software program (Stephens and Donnelly 2003; Stephens et al. 2001). Because the accuracy of statistically inferred haplotypes has been shown to increase with increasing haplotype frequency, we used polymorphisms with a minimum frequency (minor allele frequency; MAF) of 0.10 to define relatively common haplotypes (Tishkoff et al. 2000). Pairwise linkage disequilibrium (LD) was calculated as *r*^2^, a measure of the product-moment correlation coefficient (Devlin and Risch 1995).

### Resequencing: annotation of polymorphic sites.

Each gene was annotated graphically using the Artemis software program (Rutherford et al. 2000). Annotations included exon location, protein coding exon subset, reading frame, and polymorphism site. Coding region polymorphisms were evaluated for codon changes resulting from polymorphisms and the predicted effect on amino acid sequence.

### Genotyping: selection of polymorphic sites for genetic association analysis.

From the ethnicity-specific catalogs of polymorphic sites, a tagging strategy based on bins of polymorphic sites that exceeded 10% minor allele frequency and a within-bin LD (*r*^2^) exceeding 0.7 was employed, using publicly available software as recently described (Carlson et al. 2004). Because we did not know in advance the extent to which the population from Sonora would be accurately modeled in polymorphic site frequency and in LD by the EA and IA resequencing populations, tagging polymorphisms were ascertained independently in the IA and the EA populations. Tagging sites that were shared by the EA and IA groups were identified wherever possible; however, one tagging site for each bin for each group was included in the final analysis. Some nonsynonymous polymorphic sites at < 10% frequency were combined with the set of bin-tagging sites to be used in association testing.

### Genotyping: Taqman genotyping.

All polymorphic sites selected for genetic association testing were submitted for assay development to the “Assay by Design” service (Applied Biosystems). Sites that were successfully developed through this system were genotyped using the 5′-exonuclease–based Taqman assay. Reaction mixtures, consisting of 1× Taqman universal PCR master mix, 1× assay reagent (Applied Biosystems), and template (genomic DNA at 15 ng or water blank) were robotically assembled. Total reaction volume was 5 μL. Plates were sealed with optical sealing film and subjected to thermal cycling in PCR 9700 thermal cyclers (Applied Biosystems) at 95°C for 15 min, followed by 40 cycles of 92°C for 15 sec and 60°C for 1 min. Following thermal-cycling, plates were assayed for fluorescence using a 7900HT sequence detection system (Applied Biosystems). Assignment of genotypes was based on the ratio of reporter fluorescence to passive dye standard and performed automatically using the “auto-clustering” feature of the allelic discrimination software (SDS, version 2.0; Applied Biosystems).

### Genotyping: sequencing-based genotyping.

Polymorphic sites that could not be successfully designed into Taqman assays were genotyped by conventional DNA resequencing, as described above. Trained analysts scored chromatograms from each subject at the polymorphic site for the three possible genotypes.

### Genotyping: quality control.

The 22 EA samples used in the initial resequencing that produced the polymorphism catalogs were included in all genotyping samples sets, including Taqman and resequencing-based genotyping. Genotyping results from Taqman assays in the 22 EA DNA samples were compared with genotypes that were derived from resequencing in the same samples. Resequencing-based genotyping included the 22 EA subjects that were included in the original resequencing project in which the polymorphism was first identified. Deviation from Hardy-Weinberg equilibrium at each site was tested using goodness of fit chi-square or Fisher exact test. Nonassignment rates were also calculated at each site tested.

### Statistical analyses.

All statistical analyses were performed using SPSS software (version 12; SPSS, Chicago, IL, USA). Evaluation of the polymorphisms for genetic association with urinary arsenic metabolites involved calculating three derivative phenotypes: the ratio of inorganic As(III) to inorganic As(V) (3:5), the ratio of DMA(V) to MMA(V) (D:M), and the ratio of inorganic As(III) to MMA(V) (3:M). All three variables were transformed by a natural log conversion to approximate a normal distribution. Two of the phenotypes, 3:5 and D:M, were used in the initial screen for genetic association between each variable and each polymorphic site. Genotypes were recoded to conform to an analysis of a dominant genetic effect, in which the major homozygotes were assigned one categorical variable value and a second categorical variable value was assigned to the combined heterozygotes and minor homozygotes. For a given phenotype, mean values of each genotypic group were tested for difference using *t*-tests (two-tailed) for variables that did not deviate significantly from a normal distribution. Mann-Whitney and Wilcoxon tests were used to compare the means of variables that demonstrated a significant (*p* < 0.05) departure from a normal distribution, based on Kolmogorov-Smirnov or Shapiro-Wilk tests. The initial genetic association screening involved testing 23 polymorphic sites in two variables. To correct for multiple testing, a Bonferroni correction was applied to adjust each level of significance value for 46 tests. Because the presence of closely related subjects violates the assumption of the independence of samples, parents of the children that were analyzed in this study as well as one randomly selected member of each of the five sibling pairs in the study were removed from the statistical tests for difference of means between genotype groups.

We evaluated the relationship between other factors such as age, sex, daily arsenic dose (estimated by the product of arsenic well water concentration by self-reported average daily water consumption volume divided by body weight), and D:M ratio using a stepwise linear regression model, with log-transformed D:M ratio as the dependent variable and genotype, age, sex, and log-transformed daily arsenic dose as independent variables. The entry and removal criteria of the model were probabilities of *F*-values < 0.05 for entry and > 0.10 for removal. We evaluated the difference between genotype effects in adults versus children using a general linear model in a univariate analysis that included log D:M ratio as the dependent variable, with genotype and child/adult status as fixed factors, together with an interaction term for these two factors.

## Results

### *Resequencing of* CYT19.

**Figure 1:**
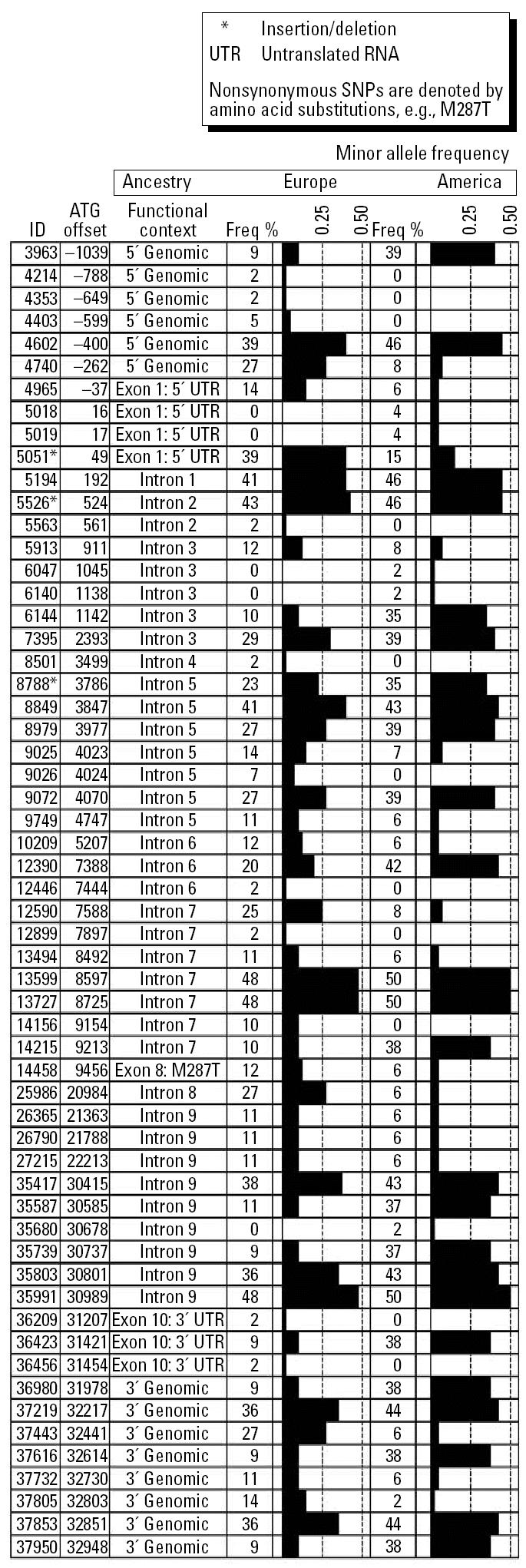
Summary of frequency and gene context of polymorphisms discovered in *CYT19* in EA (Europe) and IA (America) ancestry subjects. ID column indicates the polymorphism identification number relative to the location in the consensus sequence, with the first base of the consensus numbered 1. ATG offset column indicates the polymorphism location relative to the first base “A” of the ATG methionine initiation codon. Freq % columns are the minor allele frequency, graphically displayed in the column to the right. SNP, single nucleotide polymorphism.

In total, 12.2 kb of the genomic region that includes *CYT19* were resequenced. The polymorphic sites discovered in this effort, their frequencies and genomic contexts are displayed graphically in Figure 1. We have previously published the resequencing results for *GSTO* and *PNP* (Yu et al. 2003). The consensus sequence and the location and sequence context of all polymorphic sites in *PNP*, *CYT19*, and *GSTO* have been submitted to Genbank (accession numbers AY817667, AY817668, and AY817669, respectively) and to dbSNP (www.ncbi.nlm.nih.gov/SNP, submitter handle KLIMECKI_LAB). Because the northern Mexican population represents an admixture between IA and EA, resequencing in all genes was done in separate EA and IA populations, to obtain the largest set of sites that could be expected to be polymorphic in the subjects from Sonora. These catalogs of sites served as the basis for the selection of polymorphic sites to test for genetic association with urinary arsenic metabolite profiles. The final set of polymorphic sites selected for genetic association testing consisted of a total of 23 polymorphic sites, 5 in *GSTO*, 8 in *PNP*, and 10 in *CYT19*.

### Genotyping results.

DNA from 139 subjects was genotyped at the 23 selected sites. Initial analyses of the data involved examining the call rate on a site and a sample basis. Four DNA samples were excluded from association analysis because they failed to generate genotypes for at least 6 of the 23 sites tested. Within the remaining 135 subjects, genotype assignment rates for all sites were acceptable, with nonassignment (individual reactions in which genotypes could not be called) rates for the 23 sites ranging from 0 to 5.2%, with a mean ± SD of 2.0 ± 1.5%. No sites demonstrated statistically significant deviation from Hardy-Weinberg equilibrium. In addition to the 135 samples from the Sonoran subjects, the 22 EA samples used in the initial resequencing project that initially identified all polymorphic sites were used in all genotyping assays to allow a check of concordance. All Taqman assays were completely concordant with resequencing data. All resequencing-derived genotypes scored in the genotyping phase were completely concordant with the genotypes that were assigned in the initial resequencing of the genes.

### Genetic association analysis.

**Figure 2:**
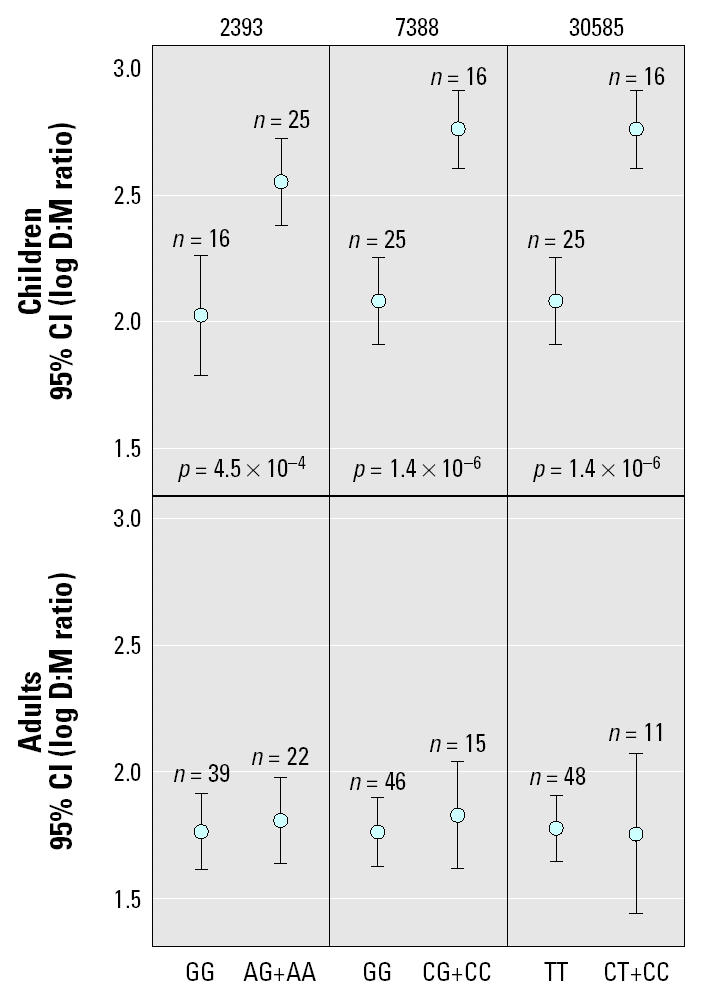
D:M ratio, stratified by genotype and age group at three *CYT19* polymorphic positions in unrelated Mexican subjects. Log-transformed D:M ratio for each group is shown as geometric mean, with error bars delineating the 95% CI of the geometric mean values. Genotype groups are depicted on the abscissa. *p*-Values (unadjusted for multiple comparisons) are from a two-tailed *t*-test comparing the geometric means of the genotype groups, and shown only when *p* < 0.05. Graphs are presented for *CYT19* sites 2393, 7388, and 30585.

**Figure 3:**
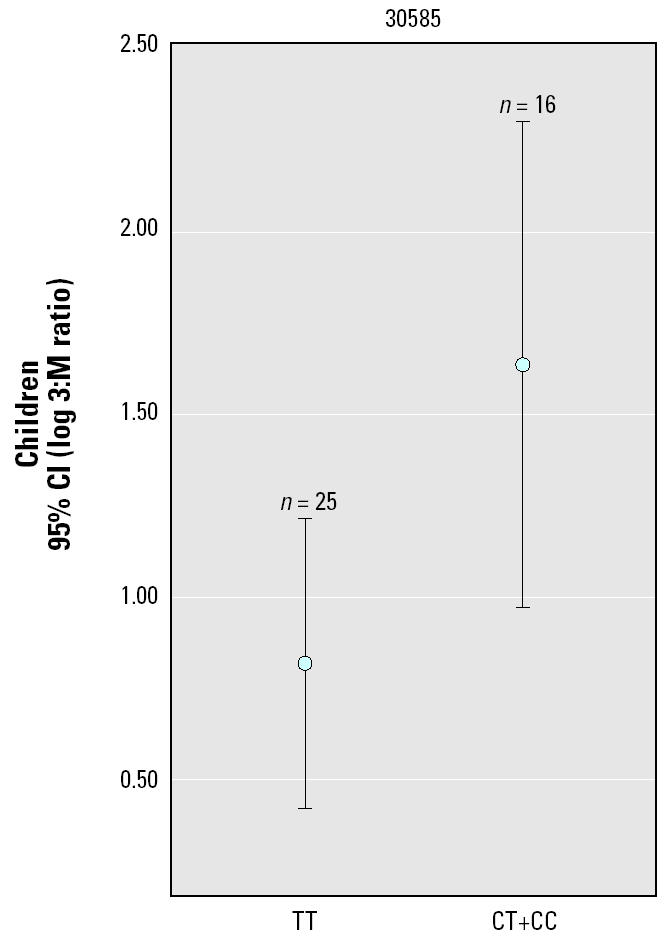
3:M ratio, stratified by genotype at *CYT19* site 30585 in the group of children analyzed in Figure 2. 3:M ratio for each genotype group is shown as geometric mean, with error bars delineating the 95% CI of the geometric mean values. Genotype groups are depicted on the abscissa. *p*-Value (unadjusted for multiple comparisons) = 0.023, two-tailed *t*-test comparing the geometric means of the genotype groups.

Table 1 summarizes characteristics, including urinary arsenic species distributions, of the study subjects. The results of the screening of these three genes for association with the 3:5 and D:M phenotypes are shown in Table 2. Testing of differences of means between the genotype groups for 3:5 used the Mann-Whitney and Wilcoxon tests because the log-transformed data did not conform to a normal distribution. Testing of means in the D:M variable used two-tailed *t*-tests. After Bonferroni correction was applied for 46 tests, three polymorphic sites in *CYT19* (2393, 7388, and 3058) were significantly associated with D:M levels in this population. To further characterize the association between *CYT19* DNA sequence and urinary D:M ratio, we tested the influence of three literature-validated potential covariates with D:M ratio, age, sex, and daily arsenic dose, together with the genotype at site 30585, which had the strongest association with D:M ratio (Chowdhury et al. 2003; Hopenhayn-Rich et al. 1996a, 1996b). This characterization was performed using a stepwise linear regression model, incorporating log-converted D:M ratio as the dependent variable, and *CYT19* 30585 genotype, age, sex, and log-converted daily arsenic dose (micrograms arsenic per kilogram body weight) as independent variables. In the final model, the only factors included were *CYT19* 30585 genotype and age, both of which were highly significant (*p* < 0.001; data not shown). We then reexamined the data in light of the strong effect of age on the relationship between *CYT19* genotype and D:M ratio. The distribution of age in this population consisted of a distinct group of children (*n* = 45) whose age ranged from 7 to 11 years. A second group consisted of adults whose ages ranged from 18 to 79 years. Given our observation of a strong age and genotype effect in D:M values, as well as studies that have reported that arsenic metabolism as measured by D:M ratio may be developmentally regulated, we stratified the population into children and adults, and tested the *CYT19* genetic association with D:M ratio separately within each group. Figure 2 shows plots of the 95% confidence intervals (CIs) of the mean log-converted D:M ratio by genotype, together with the significance values for *t*-tests comparing the genotype-grouped means, for the three *CYT19* polymorphic sites, analyzed separately for adults and children. Although no statistically significant genetic association is seen in adults, a highly significant genetic association is observed in children. Using a univariate general linear model approach to evaluate the significance of the difference between the response of children and adults, we tested age (as a dichotomous variable, child, or adult) and *CYT19* 30585 genotype as fixed factors against D:M ratio as a dependent variable. The interaction term between age and *CYT19* site 30585 genotype was highly significant (*p* = 0.0004, unadjusted for multiple comparisons), suggesting that the effect of *CYT19* genotype at site 30585 relative to D:M value is different in children from that in adults. As indicated above, the parents of the children were removed from the adult group to allow statistical testing that assumes sample independence. Nevertheless, when the mean D:M ratio by *CYT19* 30585 genotype group is compared only between children and their parents, a similar child-specific effect of the variant allele on D:M ratio is observed (data not shown). Because the D:M phenotype has a self-evident relationship with the second arsenic methylation, we created a phenotypic variable to explore the first arsenic methylation step, the 3:M ratio. Figure 3 shows the genetic association testing in this phenotype for site 30585, in which a statistically significant difference between genotypes is observed in children.

We performed LD and haplotype analysis to further characterize the occurrence of multiple polymorphism sites in strong association with D:M ratio. Pairwise LD analysis demonstrated that all three *CYT19* sites are in significant LD, with *r* ^2^ = 0.56 for sites 2393 and 30585 and *r*^2^ = 0.94 for sites 7388 and 30585. Genotypes for each child, for all 10 *CYT19* sites, were input into the Gibbs sampling-based haplotype analysis program PHASE (Stephens and Donnelly 2003; Stephens et al. 2001). The haplotypes inferred from this analysis were filtered to remove any haplotype predicted to occur on only one chromosome, because of the low confidence of these rare predictions. Five haplotypes were predicted to occur on two or more chromosomes. The variant alleles at sites 7388 and 30585 only occur together, and only on one haplotype. The variant allele at site 2393 occurs on two haplotypes, one of which contains the variant alleles for sites 7388 and 30585.

## Discussion

To our knowledge this is the most comprehensive genetic association study of arsenic metabolism to date. We observed an extremely strong association between the DNA sequence of *CYT19* and D:M ratios, an association that remained highly significant even after conservative multiple testing correction. This association was confined to the children in the study. Although the finding of a genetically and developmentally restricted association with arsenic metabolism was unexpected, the presence of a developmentally restricted component in the metabolism of arsenic has been documented (Chowdhury et al. 2003; Kurttio et al. 1998). Our observations were consistent with these reports, in that we observed an overall higher D:M ratio in children. In the study by Kurttio et al. (1998), a random effects analysis found a highly significant effect of age within the 7- to 13-year age group on DMA levels in the urine, with children of this age group having higher DMA levels. It is noteworthy that in the report by Chowdhury et al. (2003), a graph depicting age-specific D:M ratio demonstrates both a pronounced peak in D:M ratio and considerable interindividual variability, within an age range that is similar to that of the children in our study. It is possible that genetic variation in *CYT19* also explains the variability of the children’s D:M ratio values in that study.

Figure 2 also shows that the allele frequency of the positively associated polymorphic sites in *CYT19* differed between children and adults. Despite the fact that among the 23 sites examined no statistically significant difference in allele frequency between children and adults was observed (data not shown), it is possible that the allele frequency difference between children and adults may reflect real differences in the extent of admixture between children and adults. In a preliminary exploration of this, we compared the difference in allele frequency between EA and IA persons at each of the 23 polymorphic sites from the resequencing data against the allele frequency difference between children and adults from Mexico at the same sites and found a statistically significant negative correlation (data not shown), suggesting that a participant selection bias favored more IA ancestry children, more EA adults, or both. If this admixture bias is real, two potential ramifications relative to our observation of a child-specific effect must be considered. The first is that we have simply tested a marker for IA background that has nothing to do with this particular biochemical process. We feel that this is unlikely, given the magnitude of the effect and the relatively tight distribution of D:M ratios for children and adults within a scenario that would predict that the children are only marginally more “indigenous American” than the adults. Alternatively, admixture bias could possibly underlie a different LD structure in the children than in the adults. In this scenario the effect in D:M ratio that we observed would not be child specific, but rather an LD-specific effect due to our tested markers indirectly “tagging” for another marker, specifically in children. Although this scenario is possible, analysis of the pairwise LD in the existing data from the Mexican population does not support larger blocks of LD in the children compared with the adults. Even if child-specific LD was involved in the child-specific genetic association, only the location of the causal polymorphism would be potentially changed, not the underlying biologic significance of the genetic association. Although not precluding it, our data do not provide strong support for an LD-specific effect in children. Another potential confounding factor is the sex distribution between children, in which there was a similar fraction of males and females, and the adult group, which was skewed toward females. Although it is possible that this could have biased the results in the adult group away from a genetic association, we think that the marginal difference in sex composition between children and adults is unlikely to explain the difference between the sizable effect seen in children and lack of observed effect in adults.

One explanation for the presence of an overall developmental association to the phenotype, concurrent with a developmental association between genotype and phenotype, is that the same biochemical process is involved with both observations. If the widely observed developmental association between age and D:M ratio is caused by the regulation of *CYT19* expression, and the effect of the *CYT19* polymorphisms that were tested in this study occurred through modulation of *CYT19* expression, then a developmentally influenced genetic association would not be unexpected. Notwithstanding the potential that these polymorphisms may be important in adults who are arsenic-exposed under different conditions, this study provides clear evidence that in children exposed to low doses of arsenic in drinking water there exist significant genetic determinants that are strongly associated with the distribution of urinary arsenic metabolites, measured by two variables, D:M ratio and 3:M ratio. It is reasonable to generalize this to a genetic association with altered arsenic metabolism itself in children. However, because we did not measure all known metabolic intermediate species of arsenic, a more specific assignment of genetic association to particular metabolic steps awaits more detailed studies.

The actual genetic variation or variations that are the causative source of the phenotypic difference cannot be determined from the existing data. The fact that the strength of the genetic association is proportional to the LD values with the most highly associated site, 30585, raises the possibility that the source of the association signal is a cluster of polymorphic sites that is marked by site 30585. Likewise, the fact that the sites with the two strongest association signals, 7398 and 30585, perfectly define a single haplotype (they only occur together, and only on one haplotype) suggests that an effect owing to this haplotype cannot be excluded as the source of the association signal. Ultimately, resequencing the affected subjects will be required to identify the set of polymorphisms that was tagged by sites 7398 and 30585 in the association study subjects. A frequent focus of genetic association studies is nonsynonymous polymorphisms. Resequencing *CYT19* revealed only one non-synonymous polymorphism, a methionine-to-threonine substitution at residue 287, located at genomic site 9456. In both the EA and IA resequencing populations, this site was in perfect LD with *CYT19* site 5207, which we tested; we failed to detect any significant genetic association with any phenotypes. Thus, our data do not support altered amino acid sequence as the cause of the genetic association between *CYT19* and arsenic metabolism.

If, as has been well characterized in the arsenic toxicology literature, arsenic metabolism can govern the creation and removal of extremely toxic arsenic species, then these findings may suggest that a particular subset of exposed children may have increased susceptibility to arsenic toxicity by virtue of metabolism that is skewed toward enhanced accumulation of toxic species. Furthermore, the reemergence of arsenic compounds as contemporary pharmaceutical agents, most recently in the treatment of cancers, expands the translational scope of this research to potentially include human pharmacogenetics of arsenic-based therapy (Evens et al. 2004; Raza et al. 2004; Rousselot et al. 2004). In particular, this study should be considered in light of the current use of arsenical compounds in the treatment of cancer in children (Calleja and Warrell 2000; George et al. 2004; Ravindranath et al. 2004).

## Conclusion

We report a strong genetic association between polymorphisms of *CYT19* and D:M ratio in Mexican children but not in Mexican adults. Because the drinking water concentrations of arsenic in this study represent a range that includes a large number of children throughout the world, including North America as well as Central and South America, the public health and regulatory implications of this study are significant. Follow-up studies should be directed at replicating this finding and at a finer dissection of the entire arsenic metabolic pathway. Ideally, these studies will shed light on the general applicability of this finding to similar situations, as well as those involving differing exposure levels and differing genetic backgrounds of exposed individuals.

## Figures and Tables

**Table 1:** Characteristics of and urinary arsenic species distribution [μg/L; geometric mean (95% CI)] within all subjects.

	No.	Percent male	Age	As(III)	As(V)	MMA(V)	DMA(V)
Adult	90	29	38.6	7.4 (5.9–9.3)	1.6 (1.4–4.9)	3.6 (3.1–4.2)	22.6 (19.7–25.9)
Child	46	56	9.1	6.3 (4.4–9.2)	1.7 (1.4–2.0)	2.1 (1.5–2.7)	20.7 (15.8–27.0)

**Table 2:** Significance (*p*-values) of two-tailed *t*-tests for differences in mean phenotype values between genotype groups, corrected for multiple testing.

Gene	Site	D:M ratio
*GSTO*	1859	NS
*GSTO*	−1242	NS
*GSTO*	5711	NS
*GSTO*	8102	NS
*GSTO*	8147	NS
*PNP*	−1626	NS
*PNP*	−1545	NS
*PNP*	567	NS
*PNP*	2934	NS
*PNP*	3746	NS
*PNP*	5837	NS
*PNP*	6760	NS
*PNP*	7821	NS
*CYT19*	−400	NS
*CYT19*	−262	NS
*CYT19*	49	NS
*CYT19*	2393	0.024
*CYT19*	5207	NS
*CYT19*	7388	0.008
*CYT19*	7588	NS
*CYT19*	8597	NS
*CYT19*	20984	NS
*CYT19*	30585	0.003

NS, not statistically significant at *p* < 0.05. The 3:5 ratio was not significant for any gene studied.
